# Supplementary material for: Cardiac Progenitor Cell Exosomal miR-935 Protects against Oxidative Stress
Source: Cells. 2023 Sep 18;12(18):2300. doi: 10.3390/cells12182300 (PMC10528297; doi:10.3390/cells12182300)
Supplement: Supplementary file 1 [file cells-12-02300-s001.zip › cells-2522810-supplementary-figures.pdf]

Supplementary Material

Supplementary file 1. Expanded Methods & Figure legends

Expanded Methods

Cells and culture conditions

Primary Cells. Human cardiac progenitor cells (CPC) were obtained from human right atria appendage from adult donors, with no relevant cardiac pathology, and subjected to cardiac surgery with extracorporeal circulation; during the procedure, this tissue is normally discarded during cannulation. Procedures were approved by the Ethical and Research Committee of Hospital General Universitario Gregorio Marañón (HMUGM), Madrid, Spain with the corresponding patient informed consents (protocol code: 331/16, 16th January 2017). CPC were isolated from human myocardial samples by c-kit immunoselection, as described (Lauden et al., 2013). CPC were maintained and expanded as previously indicated, essentially under equivalent conditions to those used in the CAREMI clinical trial (EudraCT 2013-001358-81). Human mesenchymal stem cells (MSC) were obtained from the Inbiobank Stem Cell Bank, under specific regulations (R.D.1301/2006). Human dermal fibroblasts (HDF) were purchased from the American Type Culture Collection (Manassas, VA; cat# CRL-2097), ScienCell Research Laboratories (San Diego, CA; cat# 6300) and PromoCell (Heidelberg, Gemany; cat# C-12375 and C-12360). All cell cultures were expanded and maintained/treated (induction of oxidative damage and transfections) in the following medium: DMEM/F12 and Neurobasal medium (1:1) supplemented with 10% FBS, L-Glutamine, Penicilline-Streptomycine, B27(1X), N2 (1X), β-mercaptoethanol (50μM), ITS and growth factors (bFGF, IGF-II, EGF) as previously described (Lauden et al., 2013; Toran et al., 2017) in an atmosphere of 3% O_2/5% CO_2, which mimics physiologic

conditions and reduces the senescence evolution of the cultures. When exosome batches were intended to be obtained, cells were deprived of FBS, 24 h before conditioned medium was harvested for further exosome purification. Human MSC, HDF and mouse cardiac fibroblasts (mCF) were also maintained and expanded under optimal conditions, previously described (Toran et al., 2017), also in a 3% O_2/5%CO_2 atmosphere. Human umbilical vein endothelial cells (HUVEC) were a gift from Dr. Santos Mañes (CNB-CSIC, Madrid) and purchased (cc-2517) from Lonza (Walkersville, Maryland). HUVEC were maintained in gelatin-coated T-75 culture flasks (37ºC, 5% CO2, ≈95% humidity) using Medium 199 (Sigma-Aldrich. Madrid. Spain) supplemented with 10% FBS (Sigma-Aldrich), 10mM HEPES (Lonza), 3µg/ml endothelial cell growth supplement (ECGS, Sigma-Aldrich), 2mM glutamine (Lonza) and penicillin (100 U/ml) /streptomycin (1000 U/ml), (both from Lonza).

Transgenic mice and tamoxifen administration. Transgenic mice Bmi1CreERT/+ /Rsa26-floxedYFP (8- to 12-weeks-old; in C57BL/6 mice background) (Herrero et al., 2019). Tamoxifen (Tx) (Sigma) was dissolved in corn oil (Sigma) and mice were intraperitoneally (i.p.) injected with Tx every 24 h on 3 consecutive days (9 mg/40 g body weight). To maximize labeling, the procedure was done during 5 consecutive days. Animal studies were approved by the CNB-CSIC ethics committee and by the Division of Animal Protection of the Comunidad de Madrid (PA 56/11, PROEX 048/16). Animals were maintained and handled according to the recommendations of the CNB-CSIC institutional Ethics Committee. All animal procedures conformed to EU Directive 2010/63EU and Recommendation 2007/526/EC regarding the protection of animals used for experimental and other scientific purposes, enforced in Spanish law under Real Decreto 1201/2005.

Primary $Sca1^+Bmi1^+CD45^-$ (B-CPC) were sorted from Tx-Induced Bmi1CreERT/+ /Rosa26-floxed-YFP animals (Valiente-Alandi et al., 2015). Tx was

dissolved in corn oil and i.p. injected (103 g/g body weight). The heart was removed rapidly and retrograde-perfused under constant pressure (60 mm Hg; 37 °C, 8 min) in Ca2+-free buffer (113 mM NaCl, 4.7 mM KCl, 1.2 mM $MgSO_4$, 5.5 mM glucose, 0.6 mM KH_2PO_4, 0.6 mM Na_2HPO_4, 12 mM $NaHCO_3$, 10 mM $KHCO_3$, 10 mM Hepes, 10 mM 2,3- butanedione monoxime, and 30 mM taurine), as previously described (Herrero et al., 2019), Cardiomyocytes (CM) were pelleted by gravity (7 times, 30 min each) and the supernatant was used as a source of non-myocyte cardiac cell (Non-CM) and Bmi1-Sca1+ CD45^{-}, obtained by sorting.

When indicated, the animals were previously subjected to acute myocardial infarct (AMI) and processed 5 days after AMI. B-CPC were cultured in Iscove's modified Dulbecco's medium (Invitrogen) containing 10% FBS (ESCell FBS, Thermo Fisher Scientific. Spain), 100 IU/ml penicillin, 100 mg/ml streptomycin, and 2 mM L-glutamine (all from Thermo Fisher Scientific. Madrid. Spain), 10^3 units ESGRO Supplement (MerkMillipore. CA. USA), 10 ng/ml epidermal growth factor (Sigma) and 20 ng/ ml fibroblast growth factor (Thermo Fisher Scientific) (37 °C, 3% O_2, 5% CO_2).

Cell lines. HL-1 cells (immortalized mouse atrial hyperplastic cardiomyocyte-like cell line) were a gift from Dr. Fernández-Avilés (HMUGM, Madrid, Spain). HL-1 were cultured in gelatin/fibronectin-coated T-75 culture flasks or dishes (37ºC, 5% CO_2, ≈95% humidity). Cells were grown in Claycomb medium (Sigma-Aldrich) supplemented with 10% FBS, 2 mM L-glutamine (Invitrogen), with or without 100 µM norepinephrine, 100 U/mL penicillin and 100 g/mL streptomycin (all from Thermo Fisher Scientific. Madrid. Spain). Medium was changed every 24-48 h; when cells reached confluence (3-4 days), cultures were divided 1:3 after a brief trypsin treatment (trypsin-EDTA, Thermo Fisher Scientific). B-CPC^{IMM} is an immortalized cell line derived from B-CPC (Figure **1**); they were maintained in equivalent conditions to B-CPC.

For oxidative treatment, H_2O_2 (ThermoFisher Scientific) was freshly prepared at the indicated doses (100 M -20mM) and added to different cultures or co-cultures for 24-48 h. Cells were then recovered and evaluated in the different parameters shown.

Isolation and culture of adult mouse cardiomyocytes and non-myocyte cells

Non-myocyte cells and CM were obtained by the Langendorff method using retrograde perfusion through the aorta (Herrero et al., 2019). CM were pelleted by gravity (7 times, 30 min each) and the supernatant was used as a source of non-myocyte cardiac cell and Bmi1-Sca1+ $CD45^{-}$, obtained by sorting. When indicated the animals were previously subjected to AMI and processed 5 days after AMI. Sca1+CD45− cells were cultured in Iscove's modified Dulbecco's medium (Invitrogen) containing 10% FBS (ESCell FBS, Gibco-ThermoFisher Scientific. Waltham, MA. USA), 100 IU/ml penicillin, 100 mg/ml streptomycin, and 2 mM L-glutamine (all from Invitrogen), 103 units ESGRO Supplement (Millipore), 10 ng/ml epidermal growth factor (Sigma-Aldrich) and 20 ng/ ml fibroblast growth factor (Peprotech. London. UK) (37 °C, 3% O_2, 5% CO_2).

Supplementary Figures

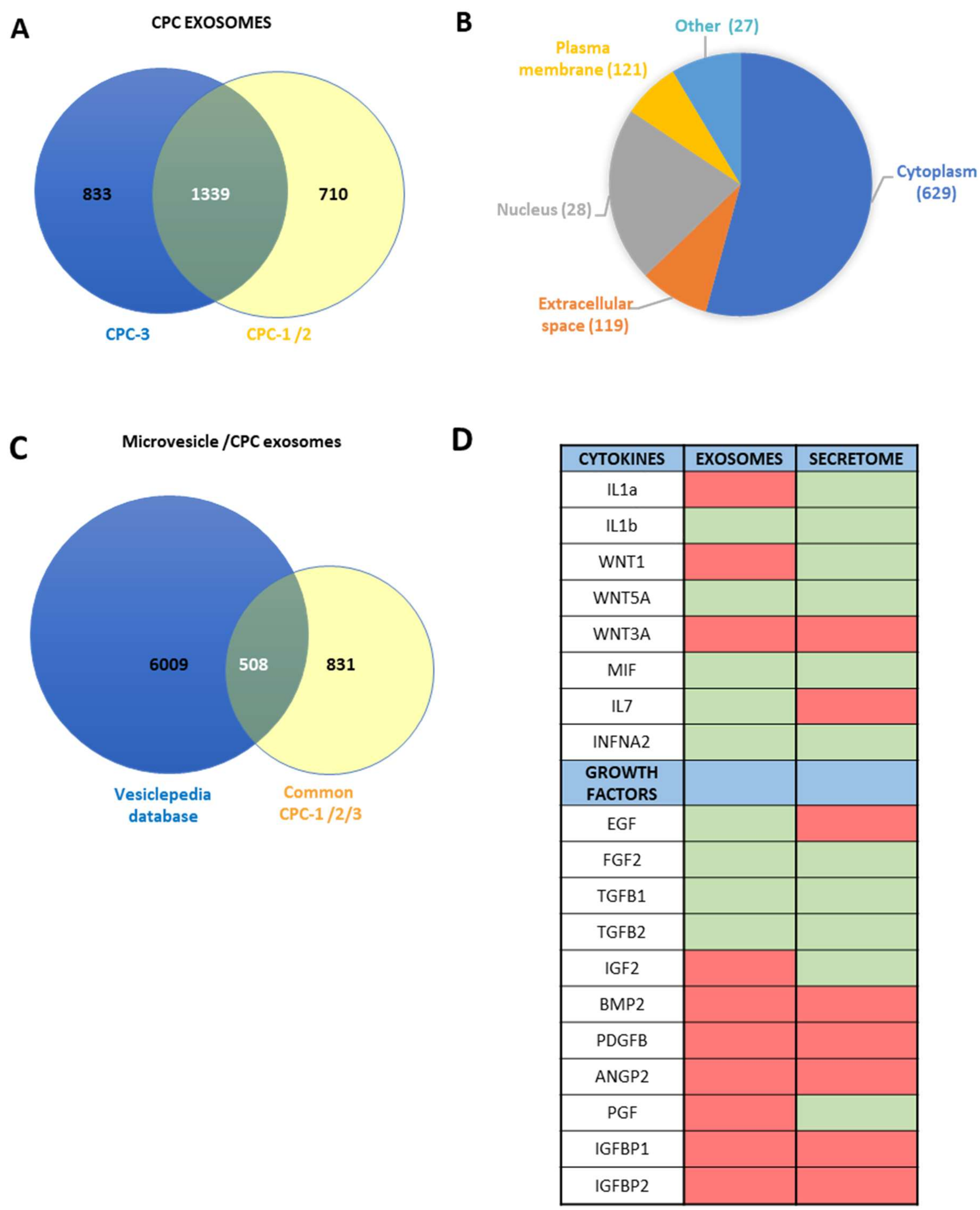

Figure S1. CPC Proteomics **(A)** Venn diagram of label-free proteomics of CPC-3 *vs.* CPC-1/CPC-2 purified exosomal fractions. Blue section indicates the proteins exclusively identified in CPC-3; yellow corresponds to proteins selectively found in CPC-1/CPC-2 and greenish, represents those proteins (1,339) commonly identified. **(B)** Venn Diagram showing the percentual representation of the main protein groups. **(C)** Comparison of CPC exosomal proteome (CPC1-3) with the microcrovesicle database); this analysis

shows that only 508 proteins of CPC exosomal proteome (greenish sector) were found in data base. **(D)** Representation of selected cytokines and growth factors in CPC exosomal compartment compared with their secretome (Toran et al. 2017).

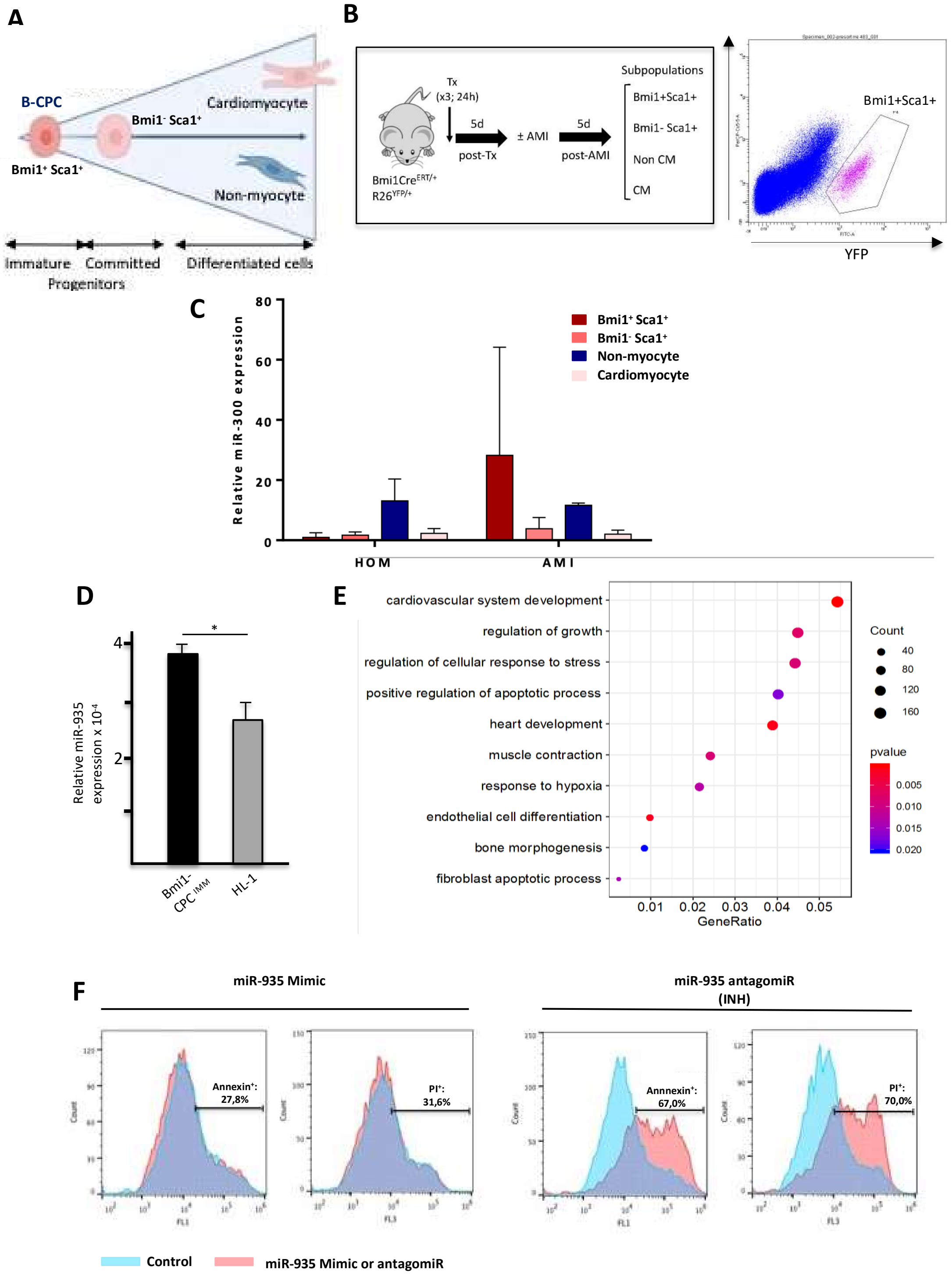

Figure S2. Evaluation of miR-935 in relevant mouse cardiac populations. (A) Scheme indicating the hierarchical relationships of the different cardiac populations analyzed: the cardiac progenitor population Bmi1⁺Sca1⁺ (B-CPC); Bmi1⁻Sca1⁺ (immediate progeny of Bmi1⁺Sca1⁺ cells); non-CM (pool of cells depleted in the CM fraction); and CM (cardiomyocyte-enriched fraction). (B) Experimental scheme (left) for isolation and

evaluation of cardiac progenitor cells (Bmi1+Sca1+) and the other resident cardiac populations (Bmi1-Sca1+; CM-cardiomyocytes and non- cardiomyocyte populations), using Tamoxifen-Induced Bmi1CreERT/+ /Rosa26-floxed-YFP animals (Herrero et al., 2019; Valiente-Alandi et al., 2015); representative histogram of the Bmi1+Sca1+ isolation (right). (C) RT-qPCR analysis of mIR-300 expression in the above indicated populations, both in homeostasis (left) and isolated post-AMI (5d). (D) Comparative miR-935 expression (RT-qPCR) between B-CPC^{IMM} and HL-1. (E) Gene Ontology (GO) analysis of the functional categories of target genes associated with the human miR-935; only categories with an enrichment p value < 0.05 were selected. (F) Cardiac fibroblasts, prior transfected (25 nM each) with miR-935 mimic, the antagomiR (INH) or the corresponding controls (scramble) were cultured during 24h and then ~~cells~~ were exposed to H_2O_2 (100 mM), during additional 24h, prior to analysis by cell cytometry, evaluating propidium iodide (PI^+) and annexin positive cells. Mimic or antagomIR transfected cells data (indicated in red) were compared to control transfected cells (greenish); percentage of PI+ or Annexing+ cells in each condition in a representative experiment are shown. (indicated in red) were compared to control transfected cells (greenish); percentage of PI+ or Annexing+ cells in each condition in a representative experiment are shown.

Figure S3. MiR-935 targets and evaluation of *in vivo* cardioprotection. **(A)** List of validated and Figure S3. bioinformatically predicted (TargetScan and miRbase database) miR-935 target genes. **(B)** HDF were transfected in homeostasis with miR-935 (mimic) or the antagomiR (INH) (25 nM each); then the cultures were maintained during 24 h and RT-qPCR of the indicated putative target genes was carried out. **(C)** RT-qPCR confirmation of Wpre expression (for viral detection) in miR935-AAV (AAV-935) intramiocardially injected mice hearts, compared with control animals analyzed at d14 (n=3 per group). **(D)** RT-qPCR evaluation of miR-935

A

	TARGET	NAME	FUNCTION	REFERENCES
VALIDATED	**NOTCH1**	NOTCH 1	Suppresses gastric signet ring cell carcinoma by targeting Notch1 expression	Yang et al., 2016
	**SOX7**	SRY-Box 7	Transcription factor. Gastric and liver cancer.<br>Promotes gastric cancer cell proliferation	Peng et al., 2018<br>Yang et al., 2016<br>Liu et al., 2017
	**INPP4A**	Inositol Polyphosphate-4 -Phosphatase Type IA	Down-regulated in pancreatic cancer	Maruyama et al., 2017
PREDICTED	**ARNT2**	Aryl Hydrocarbon Receptor Nuclear Translocator 2	Complexes with hypoxia-inducible factor1 alpha	Predicted
	**CASP10**	CASPASE 10	Apoptosis signaling; Death receptor signalling; Gap junction signalling	Predicted
	**SLC12A2**	Solute Carrier Family 12 Member 2	Aldosterone Signalling in epithelial cells. Apoptosis Signalling	Predicted
	**CSNK1G3**	Casein Kinase 1 Gamma 3	Wnt/GSK-3b in Influenza pathology	Predicted
	**RPL27A**	Ribosomal Protein L27a	EIF2 signalling	Predicted
		Hairy Ears, Y-Linked	Notch signalling	Predicted

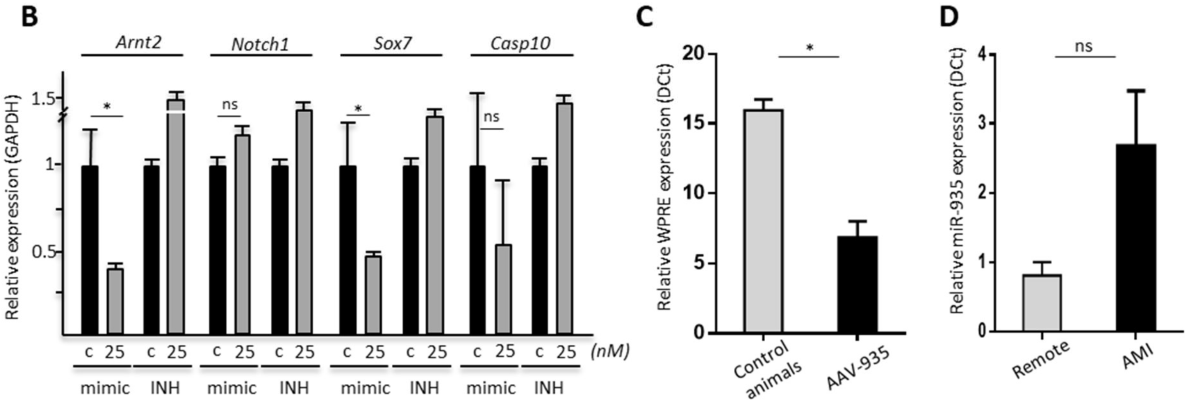

expression in infarcted mice injected with AAV-935 (n=3 per group; 60d post-injection), in the infarcted *versus* remote cardiac tissue areas.

miRNA		%CG	motifs	Exo/cell ratio in CPC
miR-2110	UUGGGGAAACGGCCGCUGAGUG	63.6	GGCC, CGCU	25×10^3
miR-1911-3p	CACCAGGCAUUGUGGUCUCC	60	UGUG // CUCC	23×10^3
miR-199a-3p	ACAGUAGUCUGCACAUUGGUUA	40.9	none	11×10^3
miR-411-5p	UAGUAGACCGUAUAGCGUACG	47.6	none	10×10^3
let-7b-5p	UGAGGUAGUAGGUUGUGUGGUU	45.5	GAGG // UGUGUG	8×10^3
let-7a-5p	UGAGGUAGUAGGUUGUAUAGUU	36.4	GAGG	6.5×10^3
mir-151a-3p	CUAGACUGAAGCUCCUUGAGG	50	CUCC // GAGG	3.5×10^3
mir-151a-5p	UCGAGGAGCUCACAGUCUAGU	52.4	GGAGC	3×10^3
mir-125a-5p	UCCCUGAGACCCUUUAACCUGUGA	52.2	UGUG	3×10^3
mir-10a-5p	UACCCUGUAGAUCCGAAUUUGUG	52.2	UGUG	2.5×10^3
				**CPC/MSC-HDF [Exos]**
miR-1268a	CGGGCGUGGUGGUGGGGG	83.3	CGGG, GGUG	Quasi-specific Exos
mir-625-3p	GACUAUAGAACUUUCCCCUCA	47.6	CCCC	7.7-fold
mir-146a-3p	CCUCUGAAAUUCAGUUCUUCAG	40.9	CCUC	3.4-fold
miR-135b-5p	UAUGGCUUUUCAUUCCUAUGUGA	34.8	UGUG, UUCC	2-fold
miR-935	CCAGUUACCGCUUCCGCUACCGC	60.9	CUUC, CGCU	no diff.
mir-146a-5p	UGAGAACUGAAUUCCAUGGGUU	40.9	UUCC	no diff.
miR-1910-3p	GAGGCAGAAGCAGGAUGACA	55	GAGG	no diff.
miR-582-3p	UAACUGGUUGAACAACUGAACC	40.9	None	no diff.
miR-31-5p	AGGCAAGAUGCUGGCAUAGCU	52.4	AGGCA	no diff.
miR-584-5p	UUAUGGUUUGCCUGGGACUGAG	50	None	no diff.
miR-149-5p	UCUGGCUCCGUGUCUUCACUCCC	60.9	UCCC	no diff.

Figure S4. Bioinformatic evaluation of the probability of miR^{SEL} exosomal miRNA to be sorted to CPC exosomes. miR^{SEL} exosomal miRNA were evaluated with respect to their exo/cell ratio and the differential expression in CPC relative to MSC/HDF. Those colored in green correspond to the exo-miRNA^{SEL} with the highest predicted probability to be sorted in CPC exosomes; miRNAs in red refer to those mainly discussed in this work.

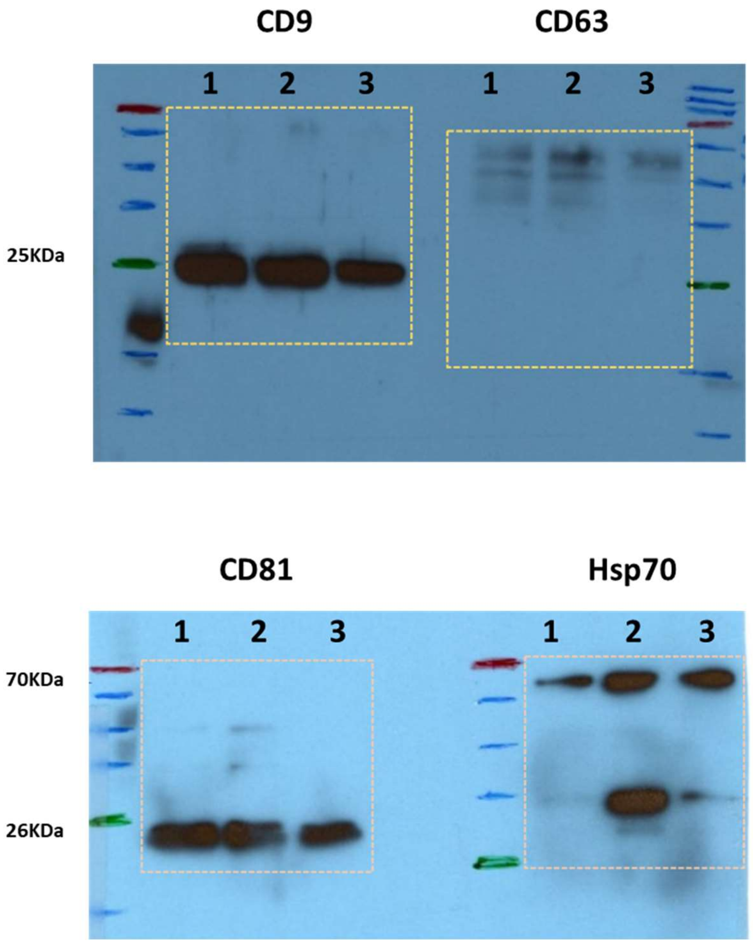

Figure S5. Full-length western blot images of sections showed in Fig. 2 (indicated here by dotted lines) for exosome preparations from different CPC isolates (CPC1-3). CD9, CD63 (top membrane), CD81 and HSP70 (bottom membrane) were evaluated with the appropriate primary goat antibodies and anti-goat-HRP as secondary antibody **(Table S2).** Protein maker samples are included and the molecular weight for the interrogated proteins is indicated.

Supplementary Tables (7):

Table S1: Oligonucleotides used in the study (.docx)

Table S2: Primary and secondary antibodies used in the study (.docx)

Table S3: Whole list of proteomic data (.xlsx)

Table S4: Comparison of miRNA representation in exosomal compartment *vs.* total CPC-3 (.xlsx)

Table S5: Differentially expressed exosomal miRNA comparing CPC with MSC_HDF (.xlsx)

Table S6: Correlation expression analysis of exosomal exo-miRSEL and transcriptional factor target genes (.xlsx)

Table S7: Main exo-miRSEL described functions in homeostasis and cancer (.xlsx).

Supplementary References

Herrero et al., 209 - [reference 32 in main text]

Lauden et al., 2013 - [reference 28 in main text]

Liu et al., 2017 - [reference 58 in main text]

Toran et al., 2017 - [reference 30 in main text]

Valiente-Alandi et al., 2015 - [reference 33 in main text]
