# Supplementary material for: Cardiac Progenitor Cell Exosomal miR-935 Protects against Oxidative Stress
Source: Cells. 2023 Sep 18;12(18):2300. doi: 10.3390/cells12182300 (PMC10528297; doi:10.3390/cells12182300)
Supplement: Supplementary file 1 [file cells-12-02300-s001.zip › Table S1. Pimers LisL Aguilar et al., 2023.pdf]

Table S1. List of primers used in this study.

Sequences

Coding Genes (RT-qPCR)	*Primer*	*Sequence (5´-3´)*
ARNT2	*FW*<br>*REV*	*GGAATGCCTACTCCAGTCTTGC*<br>*CTTTGCCACTGCGACCAGACTT*
CASP10	*FW*<br>*REV*	*CCAGGCTATGTATCCTTTCGGC*<br>*TCGTTGACAGCAGTGAGGATGG*
NOTCH1	*FW*<br>*REV*	*GGTGAACTGCTCTGAGGAGATC*<br>*GGATTGCAGTCGTCCACGTTGA*
SOX7	*FW*<br>*REV*	*TGAACGCCTTCATGGTTTGGGC*<br>*GTCAGCGCCTTCCACGACTTTC*
GAPDH	*FW*<br>*REV*	*AACTGCTTGGCACCCCTGGC*<br>*CTGGAGAGCCCCTCGGCCAT*
GUSB	*FW*<br>*REV*	*CAACGAGCCTGCGTCCCACC*<br>*ACGGAGCCCCCTTGTCTGCT*
WPRE (DNA)	*FW*<br>*REV*	*ACGCAACCCCCACTGGTT*<br>*AAAGCGAAAGTCCCGGAAAG*

miRNAs (Assay Name)	***Assay ID***	***Description (ThermoFisher Scientific)***
U6 snRNA	*001973*	*Mouse miRNA normalizer*
mmu-miR-935	*470972*	*Mouse specific miRNA-935*
hsa-miR-935	*002178*	*Human specific miRNA-935*
RNU48	*001006*	*Human miRNA normalizer*
hsa-miR30a-5p	*000417*	*Exosomal miRNA normalizer*
hsa-miR-625-3p	*002431*	*Human specific miRNA-625-3p*
mmu-miR-300-3p	*000191*	*Mouse specific miRNA-300*

Oligonucleotides for transfection	***Catalog #***	***Description (ThermoFisher Scientific)***
mmu-miR-935 Mimic	*4464066*	*mirVana® miRNA-935 mimic*
mmu-miR-935 Inhibitor	*4464084*	*mirVana® miRNA-935 inhibitor*
mmu-miR-935 Mimic Negative Control	*4464058*	*mirVana® miRNA Mimic Negative Control #1*
mmu-miR-935 Inhibitor Negative Control	*4464076*	*mirVana® miRNA Inhibitor Negative Control #1*
