# Supplementary material for: Cardiac Progenitor Cell Exosomal miR-935 Protects against Oxidative Stress
Source: Cells. 2023 Sep 18;12(18):2300. doi: 10.3390/cells12182300 (PMC10528297; doi:10.3390/cells12182300)
Supplement: Supplementary file 1 [file cells-12-02300-s001.zip › Table S2. Ac reagents. Aguilar et al., 2023.pdf]

Table S2 *List of antibodies & imaging reagents used in this study*

Western & IMM

Primary Antibody		
anti-CD9	*w*	*Ab92726 ; Abcam, Cambridge, MA, USA*
anti-CD63	*w; imm*	*Ab9269 ; Abcam, Cambridge, MA, USA*
anti-CD81	*w; imm*	*Ab79559 ; Abcam, Cambridge, MA, USA*
anti-Hsp70	*w*	*Ab78434 ; Abcam, Cambridge, MA, USA*
anti-hnRNP U	w	*Ab10297;Abcam, Cambridge, MA, USA*

Secondary Antibody		
Goat Anti-rabbit-HRP	*w*	*P0448 ; Dako, Glostrup, Denmark*
Rabbit Anti-Mouse-HRP	*w;imm*	*P0260 ; Dako, Glostrup. Denmark*
Rabbit Anti-Goat-HRP	*w; imm*	*P0449 ; Dako, Glostrup. Denmark*
Goat anti-Mouse IgG (H+L)-Fluor® 546 conjugate	*w;*	*A-11030; ThermoFisher, Spain*
