# Supplementary material for: Cardiac Progenitor Cell Exosomal miR-935 Protects against Oxidative Stress
Source: Cells. 2023 Sep 18;12(18):2300. doi: 10.3390/cells12182300 (PMC10528297; doi:10.3390/cells12182300)
Supplement: Supplementary file 1 [file cells-12-02300-s001.zip › Table S7 . ExoMIR functions. Positive and Negative implications in Cancer and Homeostasis.pdf]

Table S7 (exo-MIRs)

Homeostasis

Positive implications

miRNA (Cell/Exo)	*Functional indication*	*Target*	*Reference*
**miR-21**	Prevent cardiomyocyte apoptosis		
**-3p** *(29/1)*		PDCD4	(Xiao et al, 2016)
**-5p** *(1/1)*	Inhibit apoptosis and oxidative stress (OS)		(Young et al., 2020)
**miR-126-3p** *(1/18)*	Pro-angiogenic function in AMI	ERRFI1	(Wang et al., 2019)
	mIR-126 enriched exosomes /therapeutic against AMI		(Luo et al., 2917)
**miR-146a-5p** *(1/1)*	Attenuate oxidative stress (OS) in cardiomyocytes		(Milano et al., 2020)
	Protect against ischemia/reperfusion (Lung)	IRAK1	(Li et al., 2019)
	Protect against renal ischemia/reperfusion (Kidney)	IRAK1	(Li et al., 2020)
**miR-210** *(12/1)*	Alleviate Endothelial Oxidative Stress	Nox2	(Liu et al., 2017)
	Regulates stress-induction		(Day et al., 2019)
**miR-935** *(2/1)*	Favors prolif /different (BMSC-exo) of osteoblasts	STAT1	(Zhang et al., 2021)

Negative role

miRNA (Cell/Exo)	*Functional indication*	*Target*	*Reference*
**microRNA-27a** **-5p** *(1/1)* **-3p** *(1/1.5)*	Contribute to oxidative stress (OS)	Nrf2	(Tian et l., 2018)
**microRNA-28-3p** (7.5/1)	Contributes to oxidative stress (OS)	Nrf2	(Tian et l., 2018)
**microRNA-34a** **-5p** (11/1) **-3p** (32/0)	Contribute to oxidative stress (OS)	Nrf2	(Tian et l., 2018)
**miR-146a-5p** *(1/3)*	Aggravates Alzheimer progression; promotes neural cells	OS	(Zhan-Qiang et al., 2021)
**miR-1268a** (0/95)	Facioscapulohumeral muscular dystrophy in myotubes		(Colangelo et al., 2014)
	Gestational diabetes		(Collares et al., 2013)

Cancer

Promotion

miRNA *(Cell/Exo)*	***Functional indication***	***Target***	***Reference***
**miR-135b-5p** *(1/2)*	Promotion of gastric and pancreatic cancer	KLF4<br>NR3C2	(Liu et al., 2022)<br>(Zhang et al., 2017)
**miR-625-3p** *(1/7)*	Non-small cell lung cancer<br>Mesothelioma		(Du et al., 2020)<br>(Goricar et al., 2021)
**miR-935** *(2/1)*	Gastric cancer<br>Non-small cell lung cancer<br>Clear Cell Renal Cell Carcimoma<br>Non-small cell cancer<br>Glioma<br>Colorectal cancer<br>Colorectal cancer (generic)	SOX7<br>SOX7<br>IREB2<br>IL-27<br>HIF1α<br>INPP4A<br>KRAS-dependent sorting	(Yang et al., 2021)<br>(Peng et al., 2017)<br>(Liu et al., 2019)<br>(Abdi et al., 2020)<br>(Huang et .al., 2020)<br>(Huang et .al., 2019)<br>(Cha et al., 2015)
**miR-2110** *(0.1/10)*	Triple-negative breast cancer		(Zhang et al., 2021)

Inhibition

miRNA *(Cell/Exo)*	***Functional indication***	***Target***	***Reference***
**miR-625-3p** *(1/8)*	Gastric cancer metastasis	EZH2	(Li et al., 2020)
**miR-935** *(2/1)*	Osteosarcoma, inhibits proliferation and invasion<br>Oral Squamous Cell Carcinoma<br>Glioblastoma	HMGB1<br>INPP4A<br>FZD6	(Liu et al., 2018)<br>(Maruyama et al., 2020)<br>(Zhang et al., 2021)
